# Anti-Microbial Dendrimers against Multidrug-Resistant *P. aeruginosa* Enhance the Angiogenic Effect of Biological Burn-wound Bandages

**DOI:** 10.1038/srep22020

**Published:** 2016-02-25

**Authors:** Philippe Abdel-Sayed, Ariane Kaeppli, Thissa Siriwardena, Tamis Darbre, Karl Perron, Paris Jafari, Jean-Louis Reymond, Dominique P. Pioletti, Lee Ann Applegate

**Affiliations:** 1 Laboratory of Biomechanical Orthopedics, Institute of Bioengineering, École Polytechnique Fédérale de Lausanne (EPFL), Switzerland; 2 Department of Chemistry and Biochemistry University of Bern, Switzerland; 3 Microbiology Unit, Department of Botany and Plant Biology, University of Geneva, Switzerland; 4 Regenerative Therapy Unit, University Hospital of Lausanne, Switzerland

## Abstract

Multi-drug resistant *Pseudomonas aeruginosa* has increased progressively and impedes further regression in mortality in burn patients. Such wound infections serve as bacterial reservoir for nosocomial infections and are associated with significant morbidity and costs. Anti-microbial polycationic dendrimers G3KL and G3RL, able to kill multi-drug resistant *P. aeruginosa,* have been previously developed. The combination of these dendrimers with a class of biological bandages made of progenitor skin cells, which secrete growth factors, could positively impact wound-healing processes. However, polycations are known to be used as anti-angiogenic agents for tumor suppression. Since, neovascularization is pivotal in the healing of deep burn-wounds, the use of anti-microbial dendrimers may thus hinder the healing processes. Surprisingly, we have seen in this study that G3KL and G3RL dendrimers can have angiogenic effects. Moreover, we have shown that a dendrimer concentration ranging between 50 and 100 μg/mL in combination with the biological bandages can suppress bacterial growth without altering cell viability up to 5 days. These results show that antimicrobial dendrimers can be used in combination with biological bandages and could potentially improve the healing process with an enhanced angiogenesis.

Severe burn injuries constitute a challenging and a major public health problem, as they cause an extreme state of physiologic stress resulting in devastating metabolic responses^1^. Third-degree burns involve damage to both epidermal and dermal layers, destroying the vasculature and hindering thus spontaneous self-regeneration ability^2^. Gold standard for treatment of deep second and third degree burns is autologous skin grafting allowing to achieve wound closure^3^. However, the grafted skin is thin and more vulnerable to re-injury, which makes it less functional^4^. Moreover, auto-grafting necessitates a two-step surgical procedure, increasing total body injury with the subsequent donor site wounds for the skin grafts. When there is not enough donor skin for grafting burns, the harvesting of patient skin is also accomplished for expansion of cells in culture before the cultured skin grafts are ready for patient use (2–3 weeks) and these techniques are time and cost consuming due to respecting Good Manufacturing Processes (GMP) imposed in Hospital settings^5^.

Biological bandages have been developed to improve healing of deep degree burns, which consists of a ready-to-use cell bank of fetal progenitor skin fibroblasts made from one organ donation^6^,^7^,^8^. With one skin biopsy of 4 cm^2^, hundreds of millions of biological bandages (dimensions of 9 × 12 cm) can be prepared on native horse collagen matrices^9^. These biological bandages have several advantages, such as the ability of fetal progenitor cells to heal wounds with little inflammation involving scarless regeneration^10^. Secondly, progenitor skin cells are phenotypically stable and already differentiated involving minimal cell culture requirements such as no additional growth factors compared to other cell types^11^. Finally, skin progenitor fibroblasts can adapt and attach to all types of biomaterials, which provides efficient delivery to patients^7^. Hence, the great potential to treat burns by fetal progenitor skin cells has been shown by the application of these biological bandages, resulting in rapid and complete wound closure without auto-grafting and skin retraction, and with very little hypertrophy of the new skin^8^.

Despite the fact that biological bandages have excellent wound-healing properties they have no antimicrobial properties. Actually, the most common and severe complication in burn injuries is infection^12^. The disruption of the normal skin barrier in combination with exudate that is a protein rich environment favors microbial proliferation^13^. Furthermore, being avascular, the migration of immune cells and systematically administrated antimicrobial agents to the burn-wound are altered.

*Pseudomonas aeruginosa* has been reported to be the most frequent Gram-negative bacteria in burn wound infections and is known for being opportunistic and for developing resistance to many antibiotics^14^. Strains of multi-drug resistant (MDR) *P. aeruginosa* are frequently detected as causing nosocomial infection outbreaks in hospital burn units^15^. As such, nosocomial infections have replaced burn shock as the major cause of morbidity and mortality^16^,^17^.

Recently, it has been reported that the combination of various topology and sequence designs allowed for the discovery of potent antimicrobial peptide dendrimers (AMPDs) against MDR *P. aeruginosa*^18^. Indeed, the activities of the AMPDs, namely G3KL and G3RL (Fig. S1), were tested against four clinical isolates of *P. aeruginosa*, which were resistant to at least two different classes of antibiotics. They have shown good results with minimum inhibitory concentrations (MIC) between 4 to 32 μg/mL^18^.

Nevertheless, polycationic dendrimers are capable of having anti-angiogenic effects as shown with tumor suppression applications^19^. But for skin regeneration after deep burn wounds, angiogenesis and neovascularization are crucial factors, as newly formed blood vessels contribute to the healing process by enhancing nutrition and oxygen supply to regenerating tissues^20^,^21^. Thus, the use of polycationic AMPDs might inhibit angiogenesis and thus risk skin regeneration progress.

In the present work, we aimed to assess the possibility to use the two polycationic AMPDs, G3KL and G3RL, in combination with biological bandages for an improved treatment with anti-microbial effects on burn wounds.

## Results and Discussion

### AMPDs are not Cytotoxic and Enhance Re-Epithelialization

**Figure 1:**
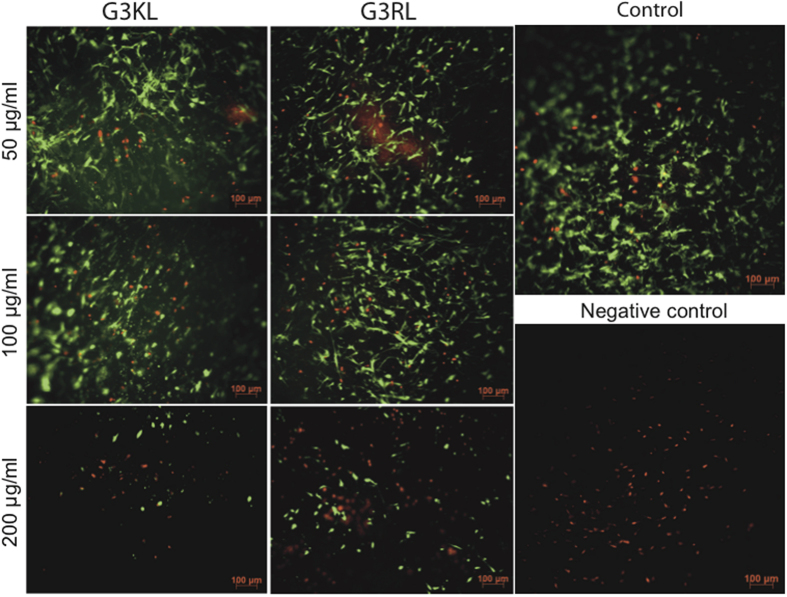
Live-Dead assay for the biological bandages after 24 h in presence of AMPDs. The living cells are shown in green and the dead cells in red. For the negative control cells were killed with methanol 30 minutes before the assay.

Firstly, we have examined the viability of the skin progenitor cells of the bandages in presence of the two dendrimers G3KL and G3RL. High dendrimer concentrations of 50, 100 and 200 μg/ml, which are one or two orders of magnitude higher than their respective MIC^18^, were tested in order to determine their cytotoxic threshold. Cell viability was determined after 24 hours of cell culture, showing a good viability of the progenitor cells to the two AMPDs at 50 and 100 μg/ml, but a significant cytotoxicity at 200 μg/ml, where the number of living cells was drastically reduced and their morphology altered (Fig. 1). Knowing that the time frame of bandage usage is usually 3–4 days, after which it is degraded and a new bandage is placed on the patient^8^, we repeated the experiment after 5 days, where good cell viability to dendrimers could still be observed for the same concentration range (Fig. S2).

**Figure 2:**
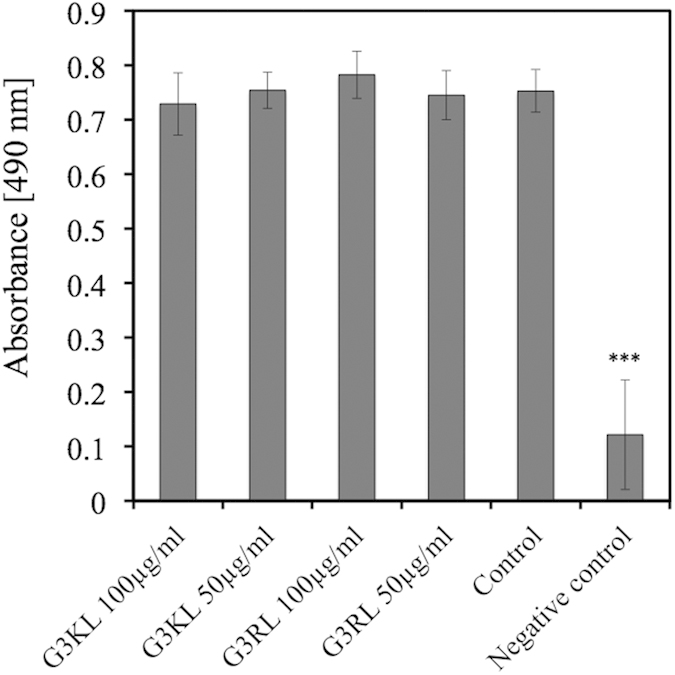
Viability of the adult fibroblasts 24 hours in presence of the dendrimers quantified with CellTiter viability Kit. Asterisks indicate statistically significant differences to positive control group (***p < 0.001, ANOVA, n = 6).

Given that AMPDs are cytotoxic at 200 μg/ml, we quantified cell viability of adult fibroblasts only at 50 and 100 μg/ml to ensure that embedded bandages at those concentrations are not harmful to patient cells. Here again, we observed no significant changes in viability between the adult fibroblasts in presence of AMPDs and the control, while the viability of the negative control was significantly reduced (Fig. 2).

**Figure 3:**
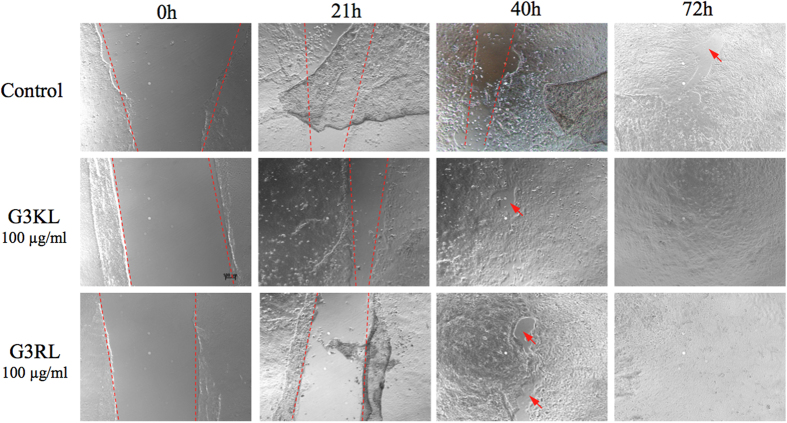
Scratch assay for visualizing keratinocyte migration for G3KL and G3RL dendrimers at 100 μg/ml over 72 hours of cell migration representing wound healing.

In order to investigate the effect of the dendrimers on wound re-epithelialization, we performed an *in vitro* scratch assay with keratinocytes and analyzed the progression of the cell migration at different time points. We observed that the presence of AMPDs at a maximum concentration of 100 μg/ml did not compromise keratinocyte migration (Fig. 3), which is essential for wound healing. We even observed a more rapid closer of the *in vitro* wound (scratch zone) in presence of the dendrimers already after 40 hours, and a complete closure after 72 hours, while a small region remained still uncovered in the control. Although the initial scratch zone is not homogenous with sharp edges it is none-the-less obvious that the cell migration was not inhibited in the presence of the maximum AMPDs concentration and the migration rate appeared to be similar in all conditions.

### AMPDs are Potent Antimicrobial Agents within the Biological Bandage Formulation

**Figure 4:**
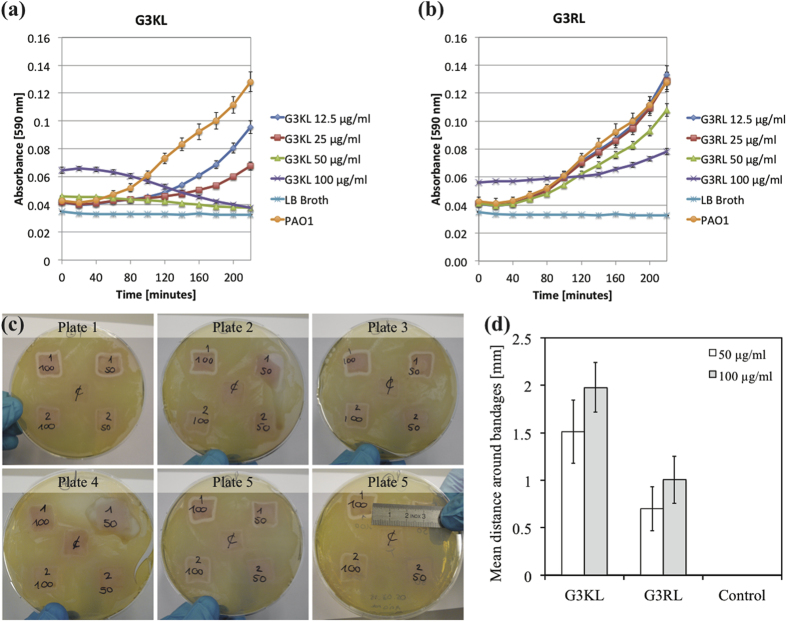
(**a**) Bacterial kinetics in presence of G3KL. (**b**) Bacterial kinetics in presence of G3RL. (**c**) Images of the zone of inhibitions: bandages noted with *1* are embedded with G3KL, those noted with *2* are embedded with G3RL and in the middle the control. 100 and 50 corresponds to their respective concentrations in μg/ml. (**d**) Mean distance of inhibition zone.

After having determined the maximum AMPD’s concentration at which cytotoxic effect is not observed, we proceeded to examine the lowest concentration at which the AMPDs could inhibit *P. aeruginosa* growth in combination with the biological bandage, and thus preventing its contamination with bacteria. Hence, an *in vitro* assay model consisting of LB broth containing *P. aeruginosa* strain PAO1 at 10^7^ CFU/mL was mixed with AMPDs solutions at different concentrations and killing kinetics were monitored by measuring the optical density (Fig. 4a,b). Actually, a bacterial load of 10^6^ CFU/ml in wound fluid is an infection threshold at which wound healing is inhibited^22^,^23^, as well as LB broth is a rich medium in nutrient favorable for bacteria growth^24^. Therefore, this model combining favorable conditions with high bacterial load (one order of magnitude than the infection threshold) ensured that an observed antimicrobial effect of the dendrimers would also be efficient in burn wound exudates. Hence, we observed for the initial 4 hours that growth curves with G3KL at 50 and 100 μg/ml were decreasing, while for lower concentrations bacteria growth was reduced in a dose response but still growing (Fig. 4a). For G3RL, the effect is less potent, as no decrease was observed except at 100 μg/ml (Fig. 4b). Remarkably, for the two AMPDs a shift was observed initially in the graph at 100 μg/ml, which is due to the high dendrimer concentration that increased the optical density of the medium. Therefore, in order to confirm that the observed antimicrobial effects at 50 and 100 μg/ml was not an optical artifact and the killing effect is not transient and can last more than 4 hours, we accomplished a zone of inhibition assay with the bandages (Fig. 4c). Biological bandages embedded with AMPDs were placed on PAO1 strains spread on LB-agar plates. The initial bacterial concentration on the plate is about 10^6^ CFU/cm^2^, which is again one order of magnitude higher than the infection load on burn wound^25^. After 24 hours, we can clearly see a zone of inhibition around the bandages that can be measured, while for the control the bandages were completely digested (Fig. 4c,d). Indeed, collagen is a great source of energy for *P. aeruginosa* by the metabolism of carbohydrates^26^, while bandages engorged with AMPDs prevent them from degradation. Furthermore, the engorgement of the bandages with AMPDs induced a fluid release with dendrimers that killed the bacteria, as clearly seen in plates 2 and 4 (Fig. 4c). Finally, the topical application of the dendrimer is not necessarily associated to collagen soaking in AMPDs solutions and the direct attachment to the collagen matrix, but can be applied separately, either directly on the wound or on the bandage, which gives freedom for the delivery system.

### AMPDs Exert direct Potent Pro-Angiogenic Effects

**Figure 5:**
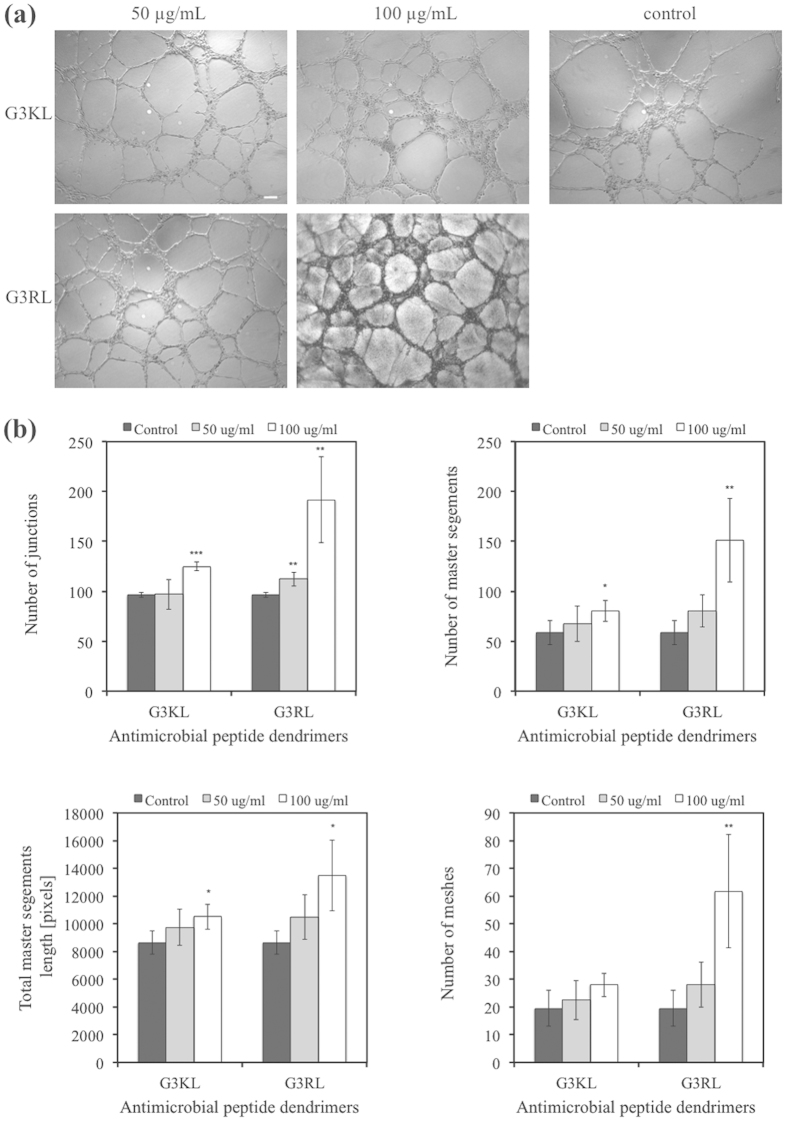
Tube formation assay. (**a**) Images of the endothelial tubular networks in Matrigel after 5 hours. (**b**) Graphs of the network characteristics. Asterisks indicate statistically significant differences to control (*p < 0.05, **p < 0.01, ***p < 0.001, paired T-test, n = 6).

We investigate the angiogenic or anti-angiogenic effect of dendrimers in order to verify that they would not alter the wound healing. For this purpose, we performed a tube formation assay with UVEC cells cultured on Matrigel in the presence of dendrimers. Tubular structures were imaged after 5 hours (Fig. 5a). We can clearly see that the endothelial tubular networks were able to develop in all the conditions and also in presence of AMPDs. Interestingly, we can see that for G3RL at 100 μg/ml, some tubules were darker, which was due to the fact that when the dendrimer mixed with the culture medium it formed a complex with another component of the medium that became more turbid. Following imaging of the complexes, we analyzed characteristic information of the networks to quantify and compare the angiogenesis potential between the groups. For G3KL and G3RL at 100 μg/ml a more evolved network in terms of number of junctions, master segments and meshes, as well as in total segment lengths could be observed (Fig. 5b).

**Figure 6:**
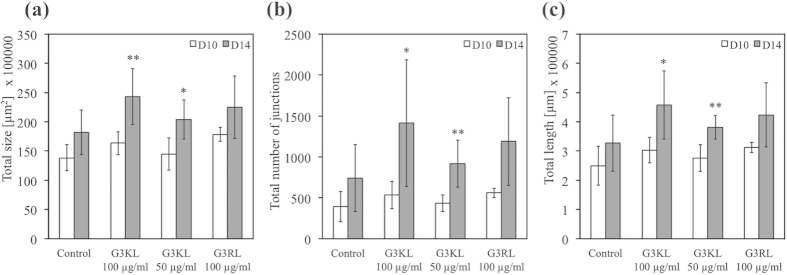
(**a**) Total surface covered by vessels. (**b**) Total number of junctions in vessels. (**c**) Total length of the vessels. Asterisks indicate statistically significant differences between day 10 and 14 (*p < 0.05, **p < 0.01, ***p < 0.001, paired T-test, n = 10–11).

In order to confirm the results of the tube formation assay, we performed then an *in vivo* CAM assay (Fig. 6). CAM assay can be used firstly as a model for *in vivo* investigation of tissue-engineered construct biocompatibility^27^ and the results of this study showed a good biocompatibility of the dendrimers at 100 μg/ml, as no adverse effects were observed. In addition, CAM assay is also used as a quantitative method for angiogenesis assessment^28^, and again our data further confirmed that G3KL at 100 μg/ml had a more developed network and thus an angiogenic effect (Fig. 6; Fig. S3). This result might appear in contradiction with what we may expect when looking in the literature, as G3KL is a poly-cation with high number of lysine molecules. Indeed, Al-Jamal and coworkers have used a cationic poly-L-lysine dendrimer as anti-angiogenic agent to delay tumor growth, and with this dendrimer they reduced significantly the neovascularization in tube formation and CAM assays for the same range of concentrations as with our study^19^. Likewise, Kasai and coworkers have designed another cationic anti-angiogenic dendrimer rich in arginine, which had a strong activity to bind to heparin to inhibit growth of vascular cells^29^. Indeed, heparin is a highly negatively charged glycosaminoglycan playing a central role in angiogenesis^30^, and which will chemically interact with any cationic dendrimer. Thus, the competition of polycations with *in situ* angiogenic factors for binding sites on heparin may induce anti-angiogenic outcomes^19^,^29^. On another hand, Pacini *et al.* have shown that poly-L-lysine with heparin stimulates angiogenesis in a CAM model^31^, which supports our results. These observations provide evidence towards the role of different dendrimer topologies and sequences that interfere differently in the electrostatic complexation of heparin with partner proteins, which in turn alter angiogenesis. Hence, not only the dendrimers charge is pivotal, but also the clustering site on heparin should be taken into consideration. This importance of heparin complexation in angiogenesis is also revealed by heparin size, where low molecular weight heparins have less affinity to pro-angiogenic partner molecules^32^,^33^.

Interestingly, it has already been shown that some heparin-binding peptides can also have antimicrobial activities^34^, and particular antimicrobial peptides can also have some angiogenic effect^35^,^36^,^37^. However, the great advantage of the dendrimers is their topology, which produces at the same time a compact and flexible globule conformation allowing a good resistance to proteolysis^38^. In general, dendrimers can be designed for several biological applications^39^, and the dendrimers of this study open a new opportunity as antimicrobial agents in the context of burn wound healing.

**Figure 7:**
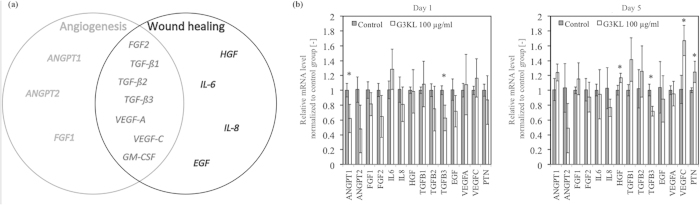
(**a**) Selected growth factors known to have an important role either in angiogenesis or in wound healing or both^43^,^44^. (**b**) Gene expression levels of human progenitor skin cells in presence of G3KL after 1 day and 5 days. Data were normalized by the housekeeping gene B2M. Cells cultured without dendrimer were used as biological control group. Asterisks indicate statistically significant differences to the control (*p < 0.05, paired T-test, n = 3).

We have shown that the dendrimer G3KL can have an angiogenic effect in a CAM assay. G3KL were associated together on the CAM membrane with progenitor cells that secrete transcription factors, which could also have improved the angiogenesis and wound healing. We asked then the following question, if the dendrimer has a direct angiogenic effect, or it simply changes the cell behavior and enhances their expression to cytokines and growth factors. For that purpose, we cultured the human progenitor skin cells in presence of the G3KL at 100 μg/ml, since it is the most potent AMPD having also better angiogenic effect in the CAM assay at this concentration. We looked at the gene expression of a number of growth factors known to have an important role in angiogenesis and/or wound healing (Fig. 7.a). The results showed that after 24 hours there was a down-regulation of ANGPT1, which is a main factor of angiogenesis, as well as in TGFB3 (Fig. 7b) Nevertheless, those results were transient variations, since after 5 days this down-regulation was not present for ANGPT1 and up-regulations for three other pro-angiogenic genes took place. Those variations in mRNA levels are less than 2-fold and most probably would not have any effect down-stream. For all the other genes, the results were not significantly altered, suggesting that most probably the two AMPDs G3KL and G3RL have a direct angiogenic effect without altering cell behavior.

In summary, we have seen that the two AMPDs G3KL and G3RL can safely be used in combination with biological bandages with concentrations up to 100 μg/ml without inducing any cytotoxic effect and also without altering the progenitor cells gene profile significantly. For the same concentration they are potent against *P. aeruginosa* with a clear zone of inhibition. Most importantly, the dendrimer G3KL in particular has shown enhanced angiogenesis in tube formation and CAM assays, which may improve the wound healing. Therefore, the topology and the sequence of dendrimers can not only affect their antimicrobial potential but can also alter their angiogenic effect.

## Methods

### Synthesis of Peptide Dendrimers

AMPDs were obtained by solid-phase peptide synthesis and purified by preparative RP-HPLC as described previously^18^. The compounds were obtained as lyophilized powders of the pure trifluoroacetate salts.

### Cell Culture and Biological Bandages Preparation

Human skin progenitor cells (FE002-SK2), adult fibroblasts (NP/JATH) and keratinocytes (immortalized HaCat) were cultured in Dulbecco’s modified Eagle’s medium (DMEM, Sigma, St. Louis, MO, USA) supplemented with 10% fetal bovine serum (FBS, Invitrogen, Carlsbad, CA, USA) and 1% L-Glutamine (Sigma). Human fetal progenitor cells were isolated from an organ donation according to a protocol approved by the State ethics committee (University Hospital of Lausanne -CHUV, Ethics Committee Protocol #62/07: organ donation, registered under the Federal Transplantation Program and its Biobank complying with the laws and regulations within both programs). Primary cells are obtained with the approval of the Ethics State Committee (Protocol #62/07) and follow regulations of the Department of Musculoskeletal Biobank. For expansion, cells were seeded at 3300 cells/cm^2^ in T75 flasks and placed in standard humidified incubators at 37 °C with 5% CO_2_, and culture medium was changed twice a week until reaching 90% confluence.

The Biological bandages were prepared as follows: Collagen TissueFleece (Baxter, Heidelberg, Germany) of 3.6 × 1.8 cm was cut in half in order to obtain squared bandages of about 1.8 × 1.8 cm. Collagen matrices were placed in 12-well plates and with tweezers four indentations were performed into the matrices. Fetal skin fibroblasts (passage 6) were suspended in PBS in order to obtain 500,000 cells/ml. 150 μl were dispensed onto each bandage in a uniform manner in order to obtain a final cells density of about 23,000 cells/cm^2^. Plates were incubated 30 minutes at room temperature to let cells to adhere to the matrix. Culture medium with/without AMPDs were then added to the bandages containing cells, which were placed in standard incubators at 37 °C.

### Cell Viability Testing with Antimicrobial Peptides

The cytotoxicity of the AMPDs was tested on the human progenitor skin cells (passage 8) cultured directly on the collagen membranes. Briefly, biological bandages were prepared as previously described, then for each AMPD 2 mg were dissolved in 10 ml culture medium in order to obtain a concentration of 200 μg/ml. Two-fold dilutions were performed to obtain AMPDs solutions of 100 and 50 μg/ml. AMPDs solutions were added to bandages and were incubated at 37 °C for 24 hours. Then, cell viability was assessed using the Viability/Cytotoxicity Assay Kit for Animal Live & Dead Cells (Biotium, Hayward, CA, USA). Reagent solutions were prepared according to manufacturer protocol, where 6 ml PBS was supplemented with 6 μl Ethidium homodimer III (EthD-III) and 3 μl Calcein AM. After washing with PBS and 45 minutes incubation of the bandages in reagent solutions, images were acquired with the microscope (Zeiss Axiovert 100, Oberkochen, Germany). For the negative control, methanol was added 30 minutes after the washing step with PBS.

Cell viability was also tested on adult fibroblasts (NP/JATH) seeded in 48-well plates at a cell density of 6,500 cells/cm^2^. AMPDs solutions were prepared as previously described for the bandages to obtain concentrations of 100 and 50 μg/ml (n = 6 wells/condition), which were added to cells 24 hours after seeding. Cell viability was measured after 24 hours using the CellTiter 96^®^ AQueous One Solution Cell Proliferation Assay Kit (Promega, Madison, WI, USA). Reagent solution was prepared according to manufacturer’s instructions. 100 μl were then distributed to each well of the plate and incubated for 1 h30. 90 μl of supernatant was placed in a new 96-well plate and the absorbance at 490 nm was measured with a spectrophotometer (Wallac Victor^2^ 1420 multilabel counter, PerkinElmer, MA, USA). For the negative control, 100 μl methanol was added 30 minutes before measurement after discarding the culture medium. Before adding the reagents used for the measurements, the culture medium of each well was aspirated and discarded and the wells were washed with 100 μl PBS (GIBCO^®^ PBS, pH 7.4 (1×), Life Technologies, Carlsbad, CA, USA).

### Scratch Assay with AMPDs

Keratinocytes (immortalized HaCat, passage 45) were seeded in 48-well plates (Cellstar, Greiner Bio One, Kremsmünster, Austria) at a density of 10,000 cells/cm^2^. Plates were placed in standard incubator at 37 °C until full confluence. The culture medium was discarded from wells and a scratch was performed vertically in each well with a 200 μl micropipette tip to create an artificial wound zone. Wells were then washed with PBS (GIBCO^®^ PBS, pH 7.4 (1X), Life Technologies, Carlsbad, CA, USA) to remove detached cells. AMPDs were dissolved in culture medium and dilutions were performed to obtain concentrations of 100 and 50 μg/ml, and finally 250 μl were dispensed in each well (n = 4 per group). Images of the scratch zones were acquired in phase contrast with a microscope (Zeiss Axiovert 100, Oberkochen, Germany) and were taken after 21, 40 and 72 h to monitor the cell migration until complete re-epitalization of the scratch zone.

### Bacterial Culture

*P. aeruginosa* PAO1 (laboratory strain) are initially stored at −80 °C in a medium composed of 50% LB (Lysogeny Broth), 40% glycerol and 10% H2O. Bacteria were retrieved from the freezer and a plastic disposable inoculating loop was immediately placed on the surface of the frozen bacteria for inoculate sampling. The bacteria were then evenly streaked on a 9-cm agar plate and incubated over night at 37 °C. A mix of colonies was picked up with an inoculating loop and distributed into tubes (14 ml Polystyrene Round-Bottom Tube, Becton Dickinson, Franklin Lakes, NJ, USA) containing 5 ml LB medium. Tubes were closed with in-hermetic caps allowing oxygen to pass and were incubated 16 h at 37 °C. The OD of the bacteria was then measured at 600 nm with a photometer (BioPhotometer Plus - Eppendorf, Hamburg, Germany). 200 μl were used to measure the OD in disposable cuvette Eppendorf® UVette® cuvette (220–1600 nm, Sigma). *P. aeruginosa* in solution were then diluted with LB in order to obtain the desired OD, knowing that OD = 1 corresponds to 1 × 10^9^ CFU/ml

### Time Kill Assay

Bacteria were cultured overnight in LB broth and diluted in order to obtain a bacteria solution of OD = 0.1, as described in above. Measurements were then performed in 96-well plates where 50 μl bacteria solution was added to 50 μl of AMPDs solutions. These later preparations were accomplished for G3KL and G3RL as previously described, to obtain AMPDs concentrations of 200 μg/ml, 100, 50 and 25 μg/ml. The 1:1 dilution in the 96-well plates with the bacteria solution gave final concentrations of 100, 50, 25 and 12.5 μg/ml for each dendrimer (n = 6 wells/condition), and a starting OD of 0.05 corresponding to 5 × 10^7^ CFU/ml. Six wells were filled with 50 μl LB broth instead of AMPD solution as control.

### Zone of Inhibition Assay

A zone of inhibition assay was performed on LB-agar plates, in order to determine the potential of the biological bandages to kill surrounding *P. aeruginosa*, on the same principle of an antibiogram test. Five conditions were tested per plate, consisting of biological bandages in combination with the two dendrimers G3KL and G3RL at two different concentrations (50 and 100 μg/ml), and one collagen matrix only as control. Technical replicates were performed (n = 5 plates) and the experiment was repeated twice. The bandages were prepared as previously described and were placed in 6-well plates containing culture medium and incubated 24 hours at 37 °C. In parallel, an overnight bacterial culture was initiated also as previously described. After the 24 hours incubation, bandages were washed with PBS containing the same corresponding AMPDs concentration, and were placed in LB agar petri dishes inoculated with 100 μl of a bacterial suspension (OD of 0.1). Plates were incubated for 24 hours at 37  °C. After incubation pictures were taken and the width of the inhibited zone without bacteria was measured around each bandage at 12 different positions (3 measurements per bandages side) with ImageJ software.

### Endothelial Cell Tube Formation Assay

Human Umbilical Vein Endothelial Cells (HUVEC) were cultured in M199 with Glutamax (GIBCO N°41150-020). For 500 ml M199, 45 mL Fetal Calf Serum (FCS), 665 μL Bovine Brain Extract at 9 mg/ml (BBE, Ruwag Handels AG), 100 μl Hydrocortisone in ethanol (5 mg/ml, Sigma), 500 μl Epidermal Growth Factor (10 μg/ml, Peprotech, London, UK) and 2.5 ml Heparin (5000 u/mL, Sigma) were added. Cells were firstly expanded until reaching 80% confluence. In parallel, 200 μl Matrigel (Corning, Bedford, MA, USA) were poured in wells of a 48-wells plate, which was then placed at 37 °C during 1 h for gel formation. Wells were washed with serum-free medium to remove extra Matrigel that did not gel. HUVECs were detached with trypsin and re-suspended in medium at a concentration of 333,000 cells/ml. 150 μl cell suspension with 150 μl peptide solution were added to each well. Plates were then placed in standard incubators at 37 °C for 5 hours, images were acquired with the microscope and the tubule-like structures in images were analyzed by the Angiogenesis Image Analyzer^40^.

### Chorioallantoic Membrane (CAM) Assay

The CAM assay was performed as follows: Initially the chicken eggs were incubated at 37 °C with the small end down. Three days later, a small hole was made in the down-side end in order to detach the membrane from the outer shell. This allowed producing a uniform larger hole on day 10 until reaching the border of the membrane. An O-ring (6 mm diameter) was deposited on the membrane and a first image of the vessels was taken. Human progenitor skin cells cultured beforehand, as well as dendrimers were then dispensed in the O-ring. Four days later, another image was taken for comparison with the image of day 10. A flowchart summarizing the different steps is illustrated in (Fig. S4). Four conditions were tested consisting of cells only as control (1), cells and G3KL at 100 μg/ml (2) and 50 μg/ml (3) and cells and G3RL at 100 μg/ml (4). Fetal skin fibroblasts were seeded in the O-ring at a density of 23,000 cells/cm^2^ corresponding to the cell density on bandages. 100 μl AMPDs solution was added on top of cells. Over the 50 eggs, 43 were viable resulting in n = 10–11 eggs per condition.

A process to binarize the images was then developed (Fig. S5). Images were then selected based on three defined criteria: (1) big vessels in the background covered more than 20% of the image, (2) the O-ring moved between the two time points and (3) the CAM membrane dried and induced artfacts. Selected binarized images were analyzed with the Matlab AngioQuant Toolbox, AngioTool and Amira softwares in order to quantify the vascularization.

### Real-Time Quantitative Reverse Transcription Polymerase Chain Reaction (qRT-PCR)

Human progenitor skin (passage 7) cells were cultured at a density of 15,000 cells/cm^2^ in 6-well plates in presence of the two antimicrobial peptides (G3KL and G3RL) at 50 and 100 μg/ml. Plates were incubated at 37 °C and 5% CO_2_. Total RNA was isolated after 24 hours and 5 days using NucleoSpin® RNA XS kit (Macherey-Nagel, Germany) according to manufacturer’s instructions. The concentration and purity of each sample were assessed by absorbance at 260 nm and by the 260/280 nm ratio, respectively with a NanoDrop Lite Spectrophotometer (Thermo Scientific, Waltham, MA, USA).

For each sample, 1 μg of total RNA was reverse transcribed using the TaqMan Reverse Transcription Reagents (Applied Biosystems), where 1 μg RNA in 19.25 μl water, 2.5 μl Random Hexamer, 5 μl Buffer 10×, 11 μl MgCl_2_, 10 μl dNTP, 1 μl Rnase Inhibitor and 1.25 μl MRTase were mixed.

Real-Time qRT-PCR was performed with custom Taqman Array Fast Plates (Applied Biosystems) according to the manufacturer’s instructions for 10 ng cDNA. B2M was used as housekeeping gene, as it was determined among four genes (18 s, GAPDH, RPII, B2M) as the most optimal normalizing gene by the NormFinder algorithm^41^. Biological and technical triplicates were performed (n = 3). The StepOnePlus Real-Time PCR system (Applied Biosystems) was used to perform the polymerase chain reaction. The data were analyzed by normalizing gene expression levels by that of the housekeeping genes^42^.

### Statistics

Data obtained were first analyzed in order to remove outliers that were statistically inconsistent with the rest of the measurements under the same condition. The modified Thompson tau technique, which is based on the mean and standard deviation of the data, was used. This method calculates a rejection zone allowing determining if a measure is an outlier or not. For CellTiter assay, an ANOVA test was then performed between the positive control and each sample with three different P-values (0.05, 0.01 and 0.001) in order to determine any significant variations. For the CAM assay, paired T-tests were performed between day 10 and 14 for each parameter and condition with three different P-values (0.05, 0.01 and 0.001) in order to determine any significant variations. For gene expression measurements, paired T-test were performed between the control and the cells cultured with G3KL at 100 μg/ml with three different P-values (0.05, 0.01 and 0.001) in order to determine any significant variations.

## Additional Information

**How to cite this article**: Abdel-Sayed, P. *et al.* Anti-Microbial Dendrimers against Multidrug-Resistant *P. aeruginosa* Enhance the Angiogenic Effect of Biological Burn-wound Bandages. *Sci. Rep.* **6**, 22020; doi: 10.1038/srep22020 (2016).

## Supplementary Material

Supplementary Information
