# Corrigendum: Anti-Microbial Dendrimers against Multidrug-Resistant *P. aeruginosa* Enhance the Angiogenic Effect of Biological Burn-wound Bandages

**DOI:** 10.1038/srep23872

**Published:** 2016-04-07

**Authors:** Philippe Abdel-Sayed, Ariane Kaeppeli, Thissa Siriwardena, Tamis Darbre, Karl Perron, Paris Jafari, Jean-Louis Reymond, Dominique P. Pioletti, Lee Ann Applegate

Scientific Reports 6: Article number: 22020; 10.1038/srep22020 Published online: 02252016; Updated: 04072016

The original version of this Article contained a typographical error in the spelling of the author Ariane Kaeppeli, which was incorrectly given as Ariane Kaeppli. This has now been corrected in the PDF and HTML versions of the Article.

