# Supplementary material for: Anti-Microbial Dendrimers against Multidrug-Resistant P. aeruginosa Enhance the Angiogenic Effect of Biological Burn-wound Bandages
Source: Sci Rep. 2016 Feb 25;6:22020. doi: 10.1038/srep22020 (PMC4766566; doi:10.1038/srep22020)
Supplement: Supplementary Information [file srep22020-s1.pdf]

SI figures

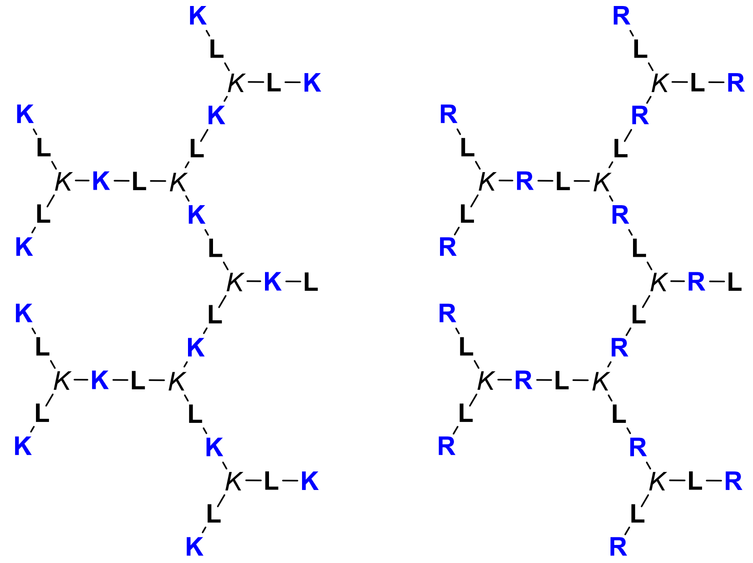

$(\mathrm{KL})_8(K\mathrm{KL})_4(K\mathrm{KL})_2K\mathrm{KL}$ $(\mathrm{RL})_8(K\mathrm{RL})_4(K\mathrm{RL})_2K\mathrm{RL}$

G3KL G3RL

Fig. S1. Molecular structures of the two AMPDs, G3KL and G3RL. K = L-lysine, R = L-arginine, *K* = branching L-lysine. The C-terminus is carboxamide and the 8 N-termini are free amines

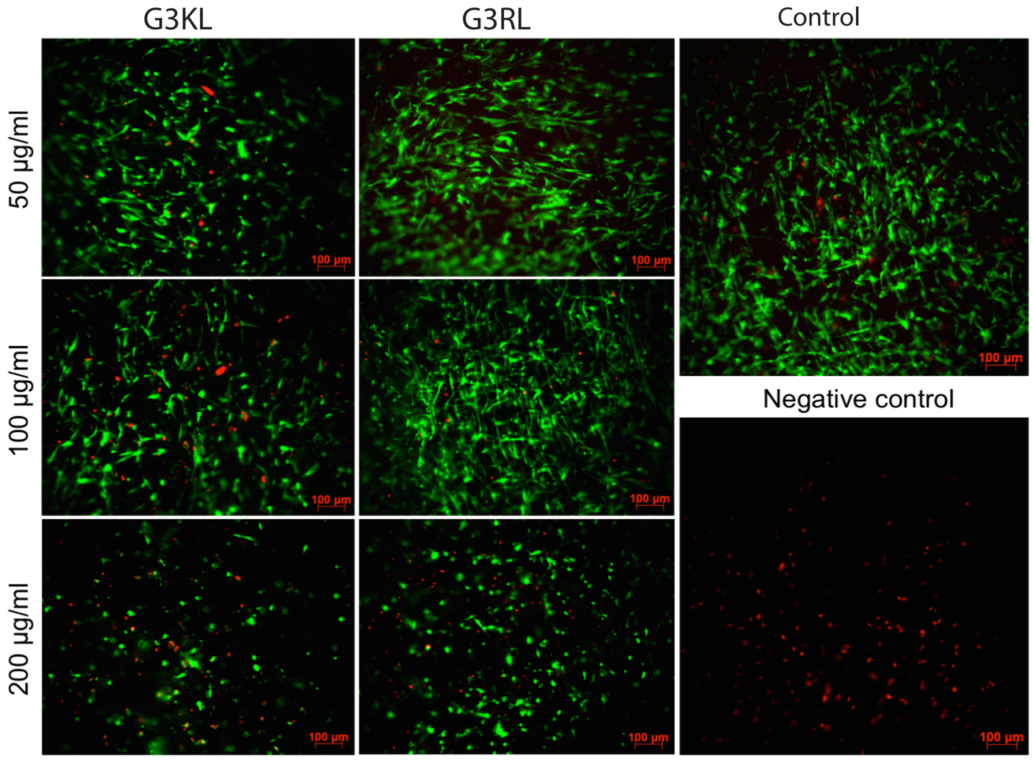

Fig. S2. Live-Dead assay for the biological bandages after 5 days in presence of AMPDs. The living cells are shown in green and the dead cells in red. For the negative control cells were killed with methanol 30 minutes before the assay.

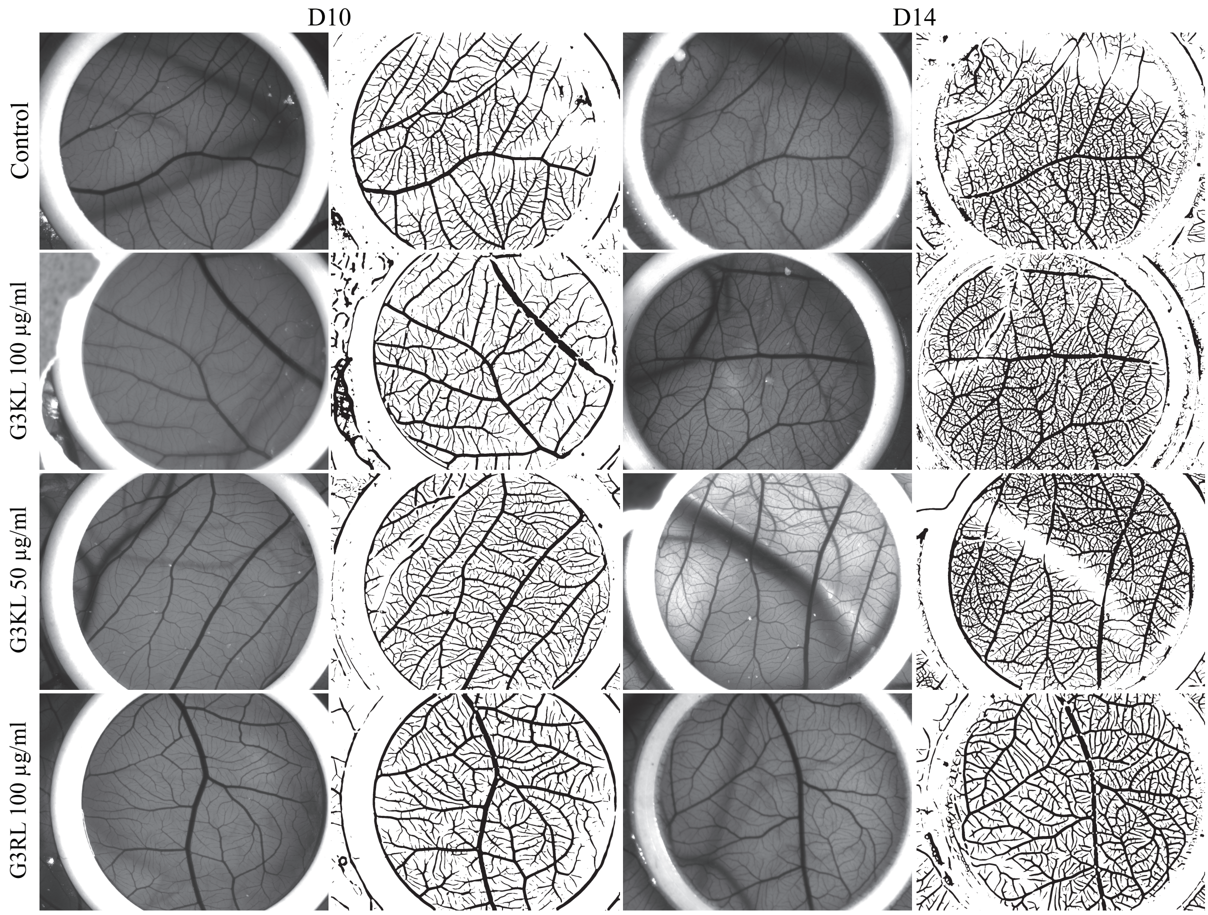

Fig. S3. Images of the CAM assay with their corresponding binarized images at day 10 and day 15.

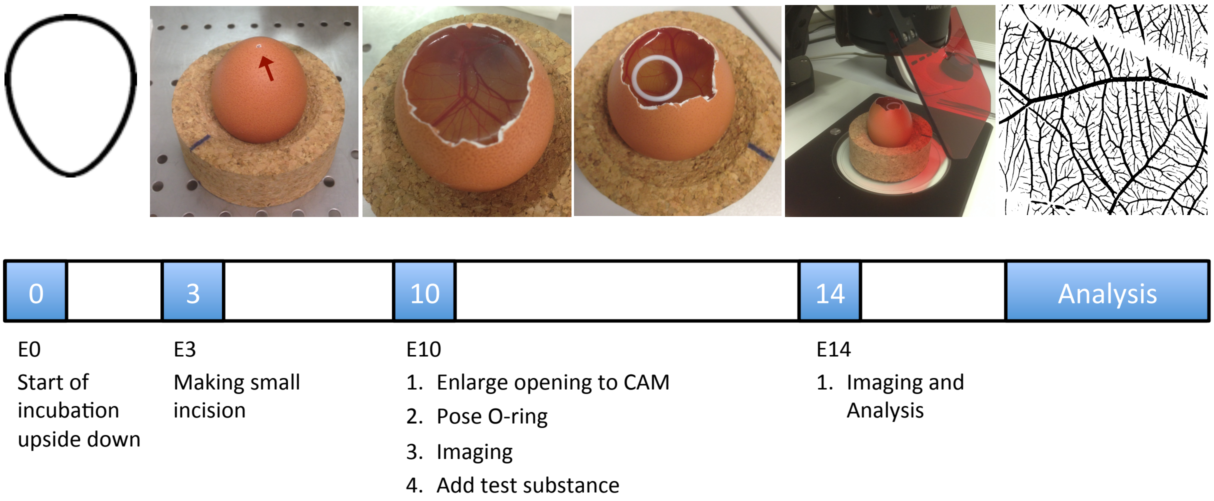

Fig. S4. Flowchart of a CAM assay experimentation.

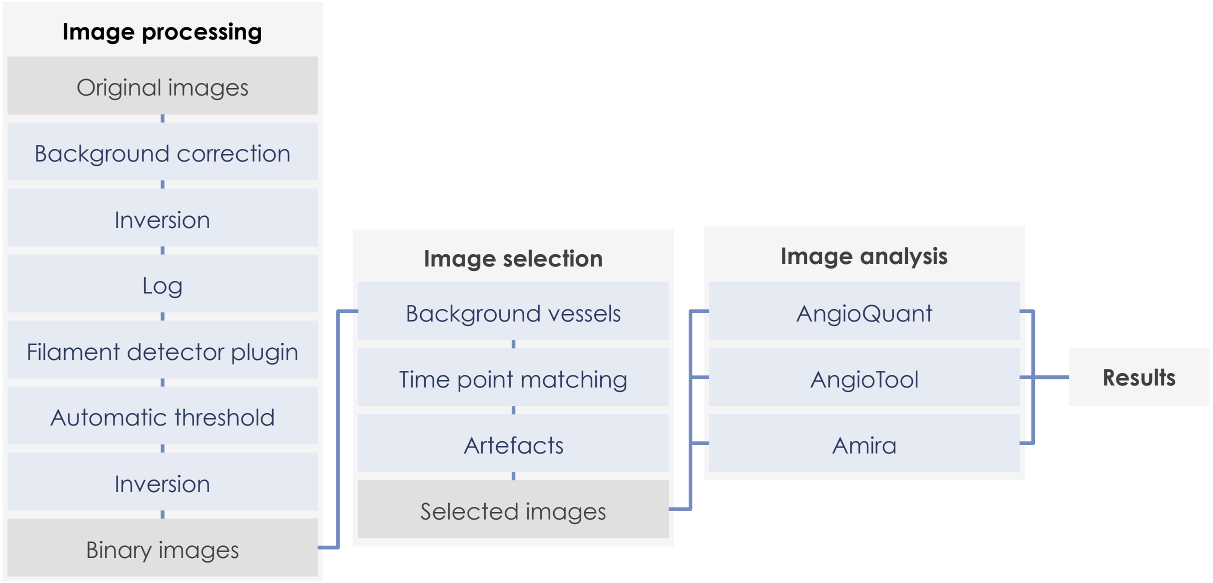

Fig. S5. Flowchart of the image processing steps in order to obtain binary images, which were then analyzed with different software.
